# High-solids enzymatic hydrolysis of ball-milled corn stover with reduced slurry viscosity and improved sugar yields

**DOI:** 10.1186/s13068-020-01717-9

**Published:** 2020-04-20

**Authors:** Minsheng Lu, Junbao Li, Lujia Han, Weihua Xiao

**Affiliations:** College of Engineering, China Agricultural University (East Campus), P.O. Box 191, 17 Qing-Hua-Dong-Lu, Hai-Dian District, Beijing, 100083 People’s Republic of China

**Keywords:** Corn stover, Ball milling, High-solids, Enzymatic hydrolysis, Rheology

## Abstract

**Background:**

High-solids enzymatic hydrolysis has attracted increasing attentions for the production of bioethanol from lignocellulosic biomass with its advantages of high product concentration, water saving, and low energy and capital costs. However, the increase of solids content would worsen the rheological properties, resulting in heat/mass transfer limitation and higher mixing energy. To address these issues, ball milling was applied to corn stover prior to enzymatic hydrolysis, and the rheological behaviors and digestibility of ball-milled corn stover under high-solids loading were investigated.

**Results:**

Ball milling significantly modified the physicochemical properties of corn stover. The apparent viscosity of slurries at 30% solid loading decreased by a factor of 500 after milling for 60 min, and the yield stress was less than 10 Pa. The dramatic decrease of viscosity and yield stress enabled the hydrolysis process to be conducted in shake flask, and remained good mixing. Meanwhile, the estimated energy consumption for mixing during saccharification decreased by 400-fold compared to the untreated one. The resultant hydrolysate using 10 FPU g^−1^ solids was determined to contain 130.5 g L^−1^ fermentable sugar, and no fermentation inhibitors were detected.

**Conclusions:**

The proposed ball milling pretreatment improved rheological behavior and sugar yield of high-solids corn stover slurry. Ball milling enables high-solids slurry to maintain low viscosity and yield stress while obtaining a non-toxic high-concentration fermentable syrup, which is undoubtedly of great significance for inter-unit processing, mixing and downstream process. In addition, the energy input for ball milling could be balanced by the reduced mixing energy. Our study indicates ball milling a promising pretreatment process for industrial bioethanol production.

## Background

Bioethanol production from lignocellulose materials is considered to be one of the solutions to improve energy structure and mitigate global climate change as its use of renewable materials such as agricultural and woody residues or energy crops. A pretreatment step using physical or chemical methods is essential to deconstruct the recalcitrance of the plant cell wall in order to increase the accessibility of cellulose and hemicellulose to enzymes. Then, the enzymes synergistically depolymerize the carbohydrates into monosaccharides for fermentation to bioethanol by microorganism. However, scale-up of the production of lignocellulosic ethanol still exist with some challenges, which impede the economic feasibility of the whole process [1–3].

A promising approach to improve the process economics is to increase the solids content in the stream. By increasing the solids content, the resulting products’ concentration will be higher, which is beneficial for reducing equipment size and energy usage for heating and distillation [4]. Running at high solids consumes less water and produces less wastewater, and therefore less input for wastewater treating. In addition, cost-effective distillation requires ethanol concentration higher than 4% (w/w), i.e., sugar concentration above 8% (w/w), which implies that for most types of lignocellulose biomass, more than 15% of solid loadings is required [5, 6]. However, increasing solids loading is not without problems, these problems may counteract the cost savings of high-solids process. For example, due to the hydroscopicity of the lignocellulose materials and low water content at high-solids condition, most of water is retained within the porous structure (cell lumen, inter-cellular space and macro/micropores), resulting in an increase of viscosity and yield stress, which could cause mixing and mass transfer problem. Inadequate mixing causes transfer problem for heat and enzymes, as well as local accumulation of products, resulting in a decrease of conversion efficiency [7]. Moreover, the power consumption of stirring is related to the viscosity, as higher impeller torque is required to overcome the shear stress under high-solid conditions [8], this would dramatically increase the power consumption of the impeller agitation and affect the process economy. Zhang et al. [9] reported that the energy consumption for mixing increased 1 order of magnitude when the solids loading of steam-exploded corn stover increased from 15 to 30% (79.5 and 1009.2 MJ t^−1^ slurry), which is equivalent to 9.3% and 58.6% of the thermal energy of ethanol produced, respectively, and attributed this to the increase of viscosity. Therefore, a better understanding of rheological behaviors of biomass slurry under high-solids loading would help to solve these challenges.

Early work by Pimenova and Hanley [10, 11] has indicated that corn stover slurries are shear thinning and yield stress fluids. The effects of solid loading, composition and particle morphology (including particle size, size distribution and aspect ratio) on the rheological properties of the biomass slurries have been reported [12–16]. Studies have also looked at the evolution of the rheological behavior during the course of saccharification or fermentation [17–19]. In general, biomass slurries containing smaller particles exhibit lower viscosity and yield stress under same solids loading [14, 15, 20], which implies that reducing particle size may potentially improve the rheology of high-solids slurry and therefore reduce the energy consumption for mixing. Ball milling is an effective method to alter the ultrastructure of lignocellulosic biomass. Our colleague showed that ball milling significantly decreases the particle size and crystallinity of rice straw, meanwhile achieving an 82.71% glucose yield by enzymatic hydrolysis (EH) using 10 FPU g^−1^ dry solids for 48 h at 5% (w/v) solids loading [21]. And ball milling can further dissociate the cross-linked cellulose–hemicellulose–lignin complex and depolymerize the cell wall polymers [22], which reduce the recalcitrance of plant cell wall and therefore may potentially improve the efficiency of EH. However, ball milling is an energy-intensive process, and a comparison between the increased energy consumption for milling and the potentially reduced mixing energy remains unknown.

The aim of this study was to investigate the effects of ball milling on the rheological properties and enzymatic hydrolysis of corn stover under high-solids loading. Corn stover was subjected to ball milling for different time, and the rheological behaviors of ball-milled corn stover (BMCS) at 30% solids loading were characterized. Meanwhile, high-solids EH of BMCS under different solids loading and enzyme loading was conducted. The changes of viscosity during EH were measured to estimate the energy consumption for stirring, and the balance between the input energy for ball milling and the reduced energy consumption for stirring was analyzed.

## Results and discussion

### Physicochemical properties of BMCS

The composition of BMCS is listed in Table 1. The results show that the carbohydrate and lignin content in the milled samples are basically the same as the unmilled one, revealing that ball milling did not change the composition of corn stover.

**Table 1:** Physicochemical properties of BMCS

	*D*_*50*_ (um)	CrI (%)	PV^a^ (cm^3^ g^−1^)	Porosity (%)	Bulk density (g cm^−3^)	Cellulose (%)	Hemicellulose (%)	Lignin (%)
BMCS0	286.58 ± 0.62	46.52	2.673	78.88	0.295	37.5 ± 0.3	22.7 ± 0.1	18.2 ± 0.2
BMCS10	75.23 ± 0.77	42.37	2.421	76.96	0.318	NM^b^	NM	NM
BMCS20	32.66 ± 0.38	26.37	1.823	71.62	0.393	NM	NM	NM
BMCS30	16.51 ± 0.52	21.03	1.187	62.05	0.523	37.2 ± 0.5	22.3 ± 0.3	18.5 ± 0.1
BMCS60	12.56 ± 0.16	9.86	0.943	56.55	0.597	NM	NM	NM
BMCS120	14.63 ± 0.26	5.04	0.935	56.22	0.602	37.1 ± 0.2	21.9 ± 0.1	18.3 ± 0.2

^a^Pore with diameter > 3 nm, i.e., mesopore and macropore (including inter-cellular space, cell lumen and some space between particles)

^b^*NM* not measured

As shown in Table 1, ball milling significantly reduced the particle size of corn stover. As ball milling time increases, the median particle size decreases sharply in the first 10 min, then slowly decreases, finally remains unchanged after 30 min. The *D*_*50*_ of BMCS0/BMCS10 is higher than 50 μm which is still at tissue scale, while the *D*_*50*_ of BMCS20/30/60/120 reaches cellular scale with particle size less than 50 μm [21, 23], indicating that the intact structure of cell wall has been destroyed.

The PV of corn stover reduced from 2.673 to 0.935 cm^3^ g^−1^, while the porosity decreased from 78.88 to 56.22%, after 120 min of ball milling (Table 1). The PV and porosity of BMCS decrease slightly at the first 10 min, then sharply decrease within 10–60 min, and finally reach a plateau. The cell lumen represents the largest scale of porosity as its size is normally in the range of tens of micrometers [24]. Results showed that pores with diameter ranging from 10 to 100 μm in BMCS0 occupy 76.0% of the total pore volume, while BMCS120 only occupies 19.2%, indicating that cell lumen occupy most of the PV of BMCS0. These results demonstrate that, at first 10 min, the intact structure of cell walls was slightly broken as the fragmentation could only reach tissue scale. Within 10–60 min, the fragmentation came to cellular scale, with a severe damage to the macropores (especially cell lumen and inter-cellular space), resulting in a sharp decrease of PV and porosity. When milling more than 60 min, there were basically no intact cell lumen, and therefore, the PV/porosity remained unchanged. These results are consistent with the previous analysis of the particle size.

**Fig. 1:**
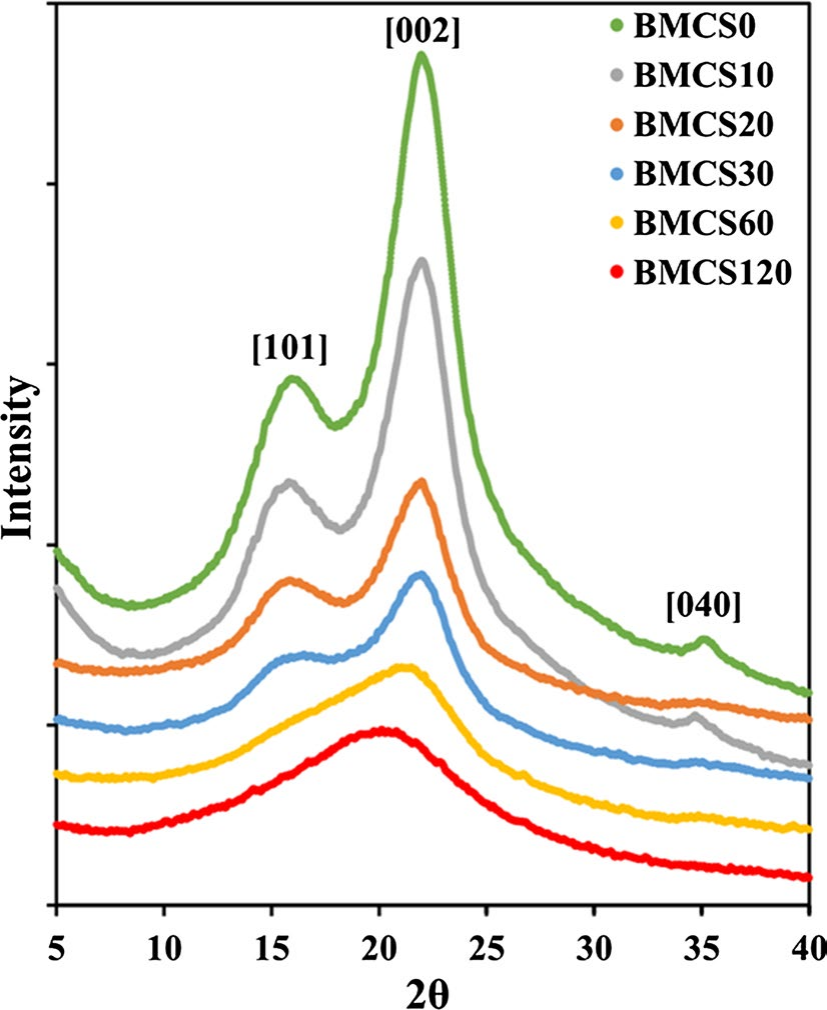
X-ray diffraction patterns of ball-milled corn stover

The XRD patterns of BMCS are shown in Fig. 1. The results demonstrate that the crystal peaks, [101], [002] and [040], gradually decrease or even disappear with the increase of milling time. The CrI determined by peak height method is listed in Table 1, and it can be found that ball milling significantly reduced the crystallinity of corn stover. The CrI of BMCS0/10 remains relatively high (46.52%/42.37%) and then drops sharply, and the CrI of BMCS120 is 5.04%, indicating the damage of ball milling goes deeper and deeper with processing time prolonging.

### Rheological behavior of BMCS slurries: apparent viscosity and yield stress

**Fig. 2:**
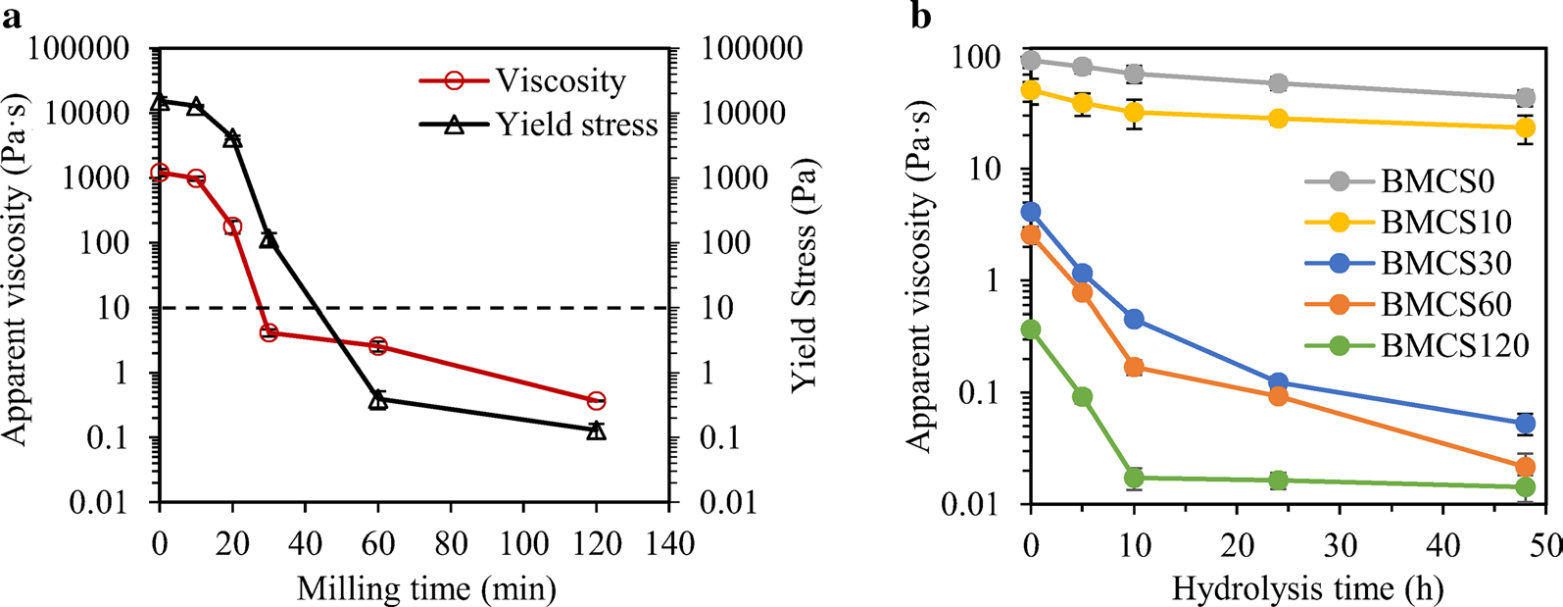
Rheological parameters at 30% solids loading **a** as a function of milling time, the horizontal-dashed line indicates the yield stress threshold (10 Pa) below which the slurry is pourable and pumpable; **b** at different enzymatic hydrolysis time, the solids loading of BMCS0 and BMCS10 is 20%. Apparent viscosity at a shear rate of 25.12 s^−1^ was used

For traditional thermochemical pretreated corn stover, the upper limit of solids content that can be effectively mixed in a conventional stirred tank reactor is 12–15% [25], and EH at 30% solids loading is relatively difficult to achieve at laboratory scale. However, we found that the BMCS slurries remained fluidity at up to 30% solids loading. Therefore, the rheological behaviors of BMCS slurries at 30% solids loading were measured within a logarithmically increasing shear rate between 0.01 and 100 s^−1^. The apparent viscosity varied greatly at different shear rates, exhibiting shear thinning behavior (see Additional file 1: Figure S1, for more details). For better comparison, the apparent viscosity at *γ*_eff_ = 25.12 s^−1^ was selected and plotted as a function of milling time, and the results are shown in Fig. 2a. It can be seen that the apparent viscosity and yield stress decrease dramatically with increasing milling time. For example, ball milling for 30 min reduced the apparent viscosity of slurry at 30% solids loading by about 300 times, further extending milling time to 120 min only reduced by around 10 times. According to the analysis in “Methods” section, the significant decrease of viscosity might have potential benefit for reducing the mixing energy during high-solids EH, this will be discussed later. It can be found that the yield stress of BMCS60/BMCS120 is less than 10 Pa (Fig. 2a). 10 Pa is considered to be a critical value below which the slurry behaves as a pourable liquid and could be easily pumped between different process units [18]. Previous studies reported a yield tress of about 1000 Pa for dilute acid-pretreated corn stover at 20% solids loading [18] and of around 1500 Pa for untreated and dilute acid-pretreated corn stover at 30% solids loading [14], which are higher than BMCS by several orders of magnitude. The dramatic decrease in apparent viscosity and yield stress may be attributed to the following two aspects. The entanglement between particles alleviated due to the significant decrease of particle size, thus reduced the apparent viscosity and yield stress [14, 20]. Another factor was the increased free water amount in slurries due to the decreased porosity (see Fig. 5 and Additional file 1: Table S1, for more details), which increased the lubrication between particles and reduced friction, thereby reduced the apparent viscosity and yield stress [14].

Figure 2b presents the changes in apparent viscosity as enzymatic hydrolysis proceeds for corn stover slurry with 30% solids loading. The apparent viscosity decreased as the hydrolysis process progressed. The decrease of viscosity could be explained by the decreased insoluble solid content and the modified particle properties as hydrolysis progressed [18, 19]. The viscosity during EH was used to estimate the energy consumption for mixing according to the theory in “Methods” section, and this is discussed in the next section.

### Energy balance for ball milling and mixing

**Fig. 3:**
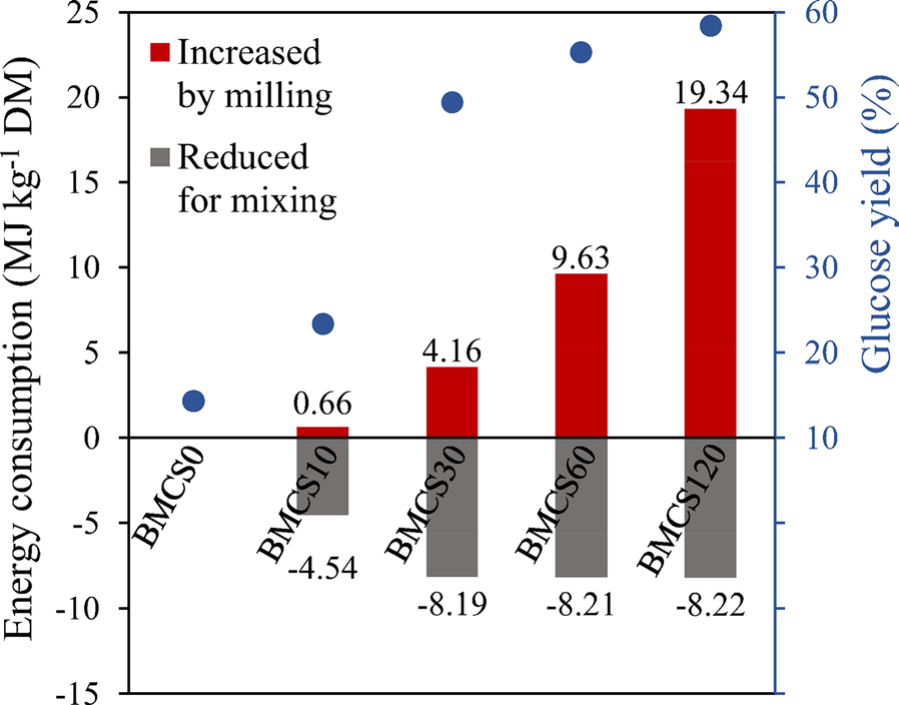
Energy balance between ball milling and mixing. The reduced energy consumption for mixing was calculated based on BMCS0, for example, BMCS30: 0.037 − 8.23 = − 8.19

The energy consumption of ball milling and the required mixing energy during EH are shown in Table 2. The results demonstrate that the energy required for ball milling increases with increasing milling time, and the energy input for BMCS120 reaches 19.34 MJ kg^−1^ DM (dry matter). On the contrary, the required mixing energy during high-solids EH decreases with increasing milling time, due to the dramatic decrease of viscosity after ball milling. The mixing energy consumption of BMCS0 is 8.23 MJ kg^−1^ DM, which is higher than that of the thermochemical pretreated biomass (Table 2) [8, 9, 26, 27]. This could be attributed to the higher viscosity, and the lower liquefaction rate (i.e., EH rate) and digestibility of untreated corn stover (BMCS0). In fact, a strict comparison between energy consumption during EH for different pretreated substrates is difficult because different stirring tank, stirring speed, solid loading and hydrolysis time all affect the energy consumption for mixing. In this study, after milling for 10 min, the energy consumption for mixing was reduced to 3.69 MJ kg^−1^ DM. And further prolonging the milling time to 30 min, the mixing energy was sharply decreased to 0.037 MJ kg^−1^ DM. To calculate the energy consumption balance between ball milling and mixing, the increased energy due to ball milling and the reduced mixing energy due to decreased viscosity based on BMCS0 were compared, and the results are shown in Fig. 3. The results show that when the milling time is less than 30 min, the increased energy consumption for milling is less than the reduced mixing energy. For example, the increased energy consumption of BMCS30 is 4.16 MJ kg^−1^ DM, while the reduced mixing energy (based on BMCS0) is 8.19 MJ kg^−1^ DM. However, the increased energy consumption could not be offset by the reduced mixing energy when prolonging milling time to 60 min or 120 min. In terms of glucose yield, the results show that the glucose yield increases to 23.3%, 49.4% and 55.3% for BMCS10, BMCS20 and BMCS30, respectively. For BMCS60, the increased energy consumption for milling is basically the same as the reduced mixing energy, while the glucose yield increased by 287% (based on BMCS0). Increasing the milling time to 120 min obtains limited improvement in glucose yield, but doubles the energy consumption of milling.

**Table 2:** Energy consumption of ball milling, and mixing energy during high-solids EH

Substrate	Milling energy (MJ kg^−1^ DM)	Reactor	Hydrolysis conditions	Solids loading (w/w)	Mixing energy (MJ kg^−1^ DM)	Glucose yield (%)	Refs.
Steam-exploded CS	NA	5 L, helical impeller	7 FPU g^−1^ DM, 72 h, 120 rpm	20%	0.57	68 (ethanol)	[9]
				25%	1.36	64.8 (ethanol)	
				30%	3.36	52.1 (ethanol)	
Steam-exploded spruce	NA	2.5 L, blade impeller	20 FPU g^−1^ glucan, 96 h, 300 rpm	10%	~ 1.7	~ 58	[26]
SO_2_ + steam-exploded spruce	NA	3 L, anchor impeller	0.1 g CTec2 g^−1^ DM, 48 h, 10 rpm	10%	0.116	~ 34	[8]
				15%	0.135	~ 28	
				20%	0.272	~ 24	
Steam-exploded sugarcane bagasse	NA	3 L, dual-impeller	10 FPU g^−1^ DM, 96 h, 470 rpm	10% (w/v)	3.2–7.53	~ 60–75	[27]
BMCS0	0	7 L, helical impeller	10 FPU g^−1^ DM, 48 h, 55 rpm	20%^a^	8.23	14.3	This study
BMCS10	0.66			20%^a^	3.69	23.3	
BMCS30	4.16			30%	0.037	49.4	
BMCS60	9.63			30%	0.021	55.3	
BMCS120	19.34			30%	0.003	58.4	

*NA* not applicable

^a^ BMCS0 and BMCS10 with 30% solids loading was hard to handle, so the data at 20% were used instead

Ball milling is considered to be an economically unfeasible pretreatment method because of its high energy consumption [28–30]. However, when the EH process is running at high solids, ball milling could significantly reduce the viscosity of the slurry, thus reducing the mixing energy and offset part of the energy consumption for milling, which is of positive implications for the feasibility of ball milling as a pretreatment method. The results also highlight the importance of considering the mixing energy consumption when conducting high-solids EH.

### High-solids enzymatic hydrolysis

Given that ball milling could significantly reduce the viscosity and yield stress of the high-solid slurry, and made the slurry meet the industrial requirements of pourability and pumpability. We then evaluated the digestibility of BMCS at high-solids loading. Because BMCS0 and BMCS10 behaved as wet granules like material at 20% and 30% solids loading, and the liquefaction was insufficient, which made sampling difficult, therefore, sugar data under these conditions could not be obtained.

**Fig. 4:**
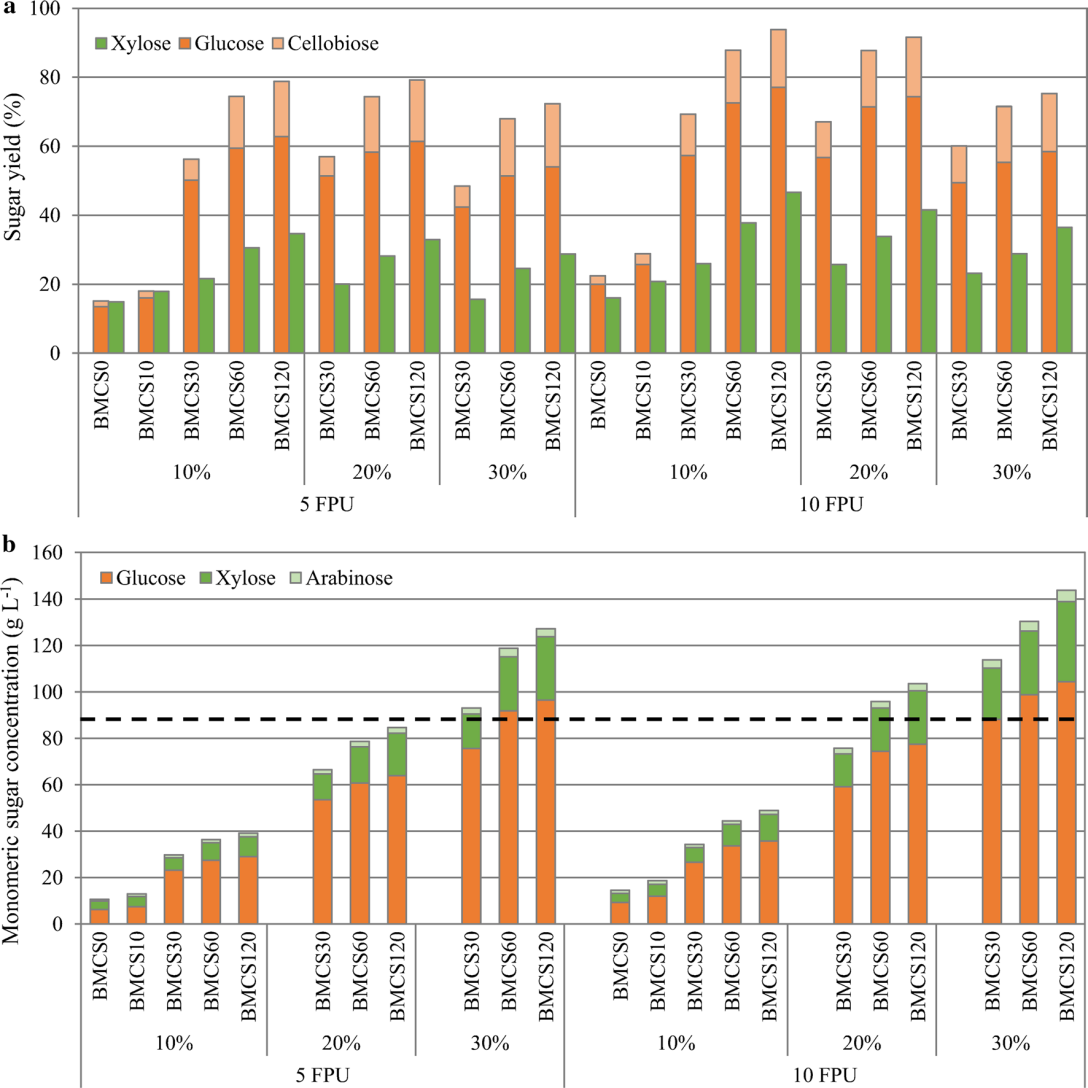
Enzymatic hydrolysis of BMCS at different solids loading with different enzyme loading. Results for **a** sugar yield and **b** monomeric sugar concentration. The horizontal-dashed line indicates the monomeric sugar concentration threshold (87 g/L) above which the distillation of ethanol is cost-effective (assume an ethanol yield of 0.5 g/g glucose)

**Fig. 5:**
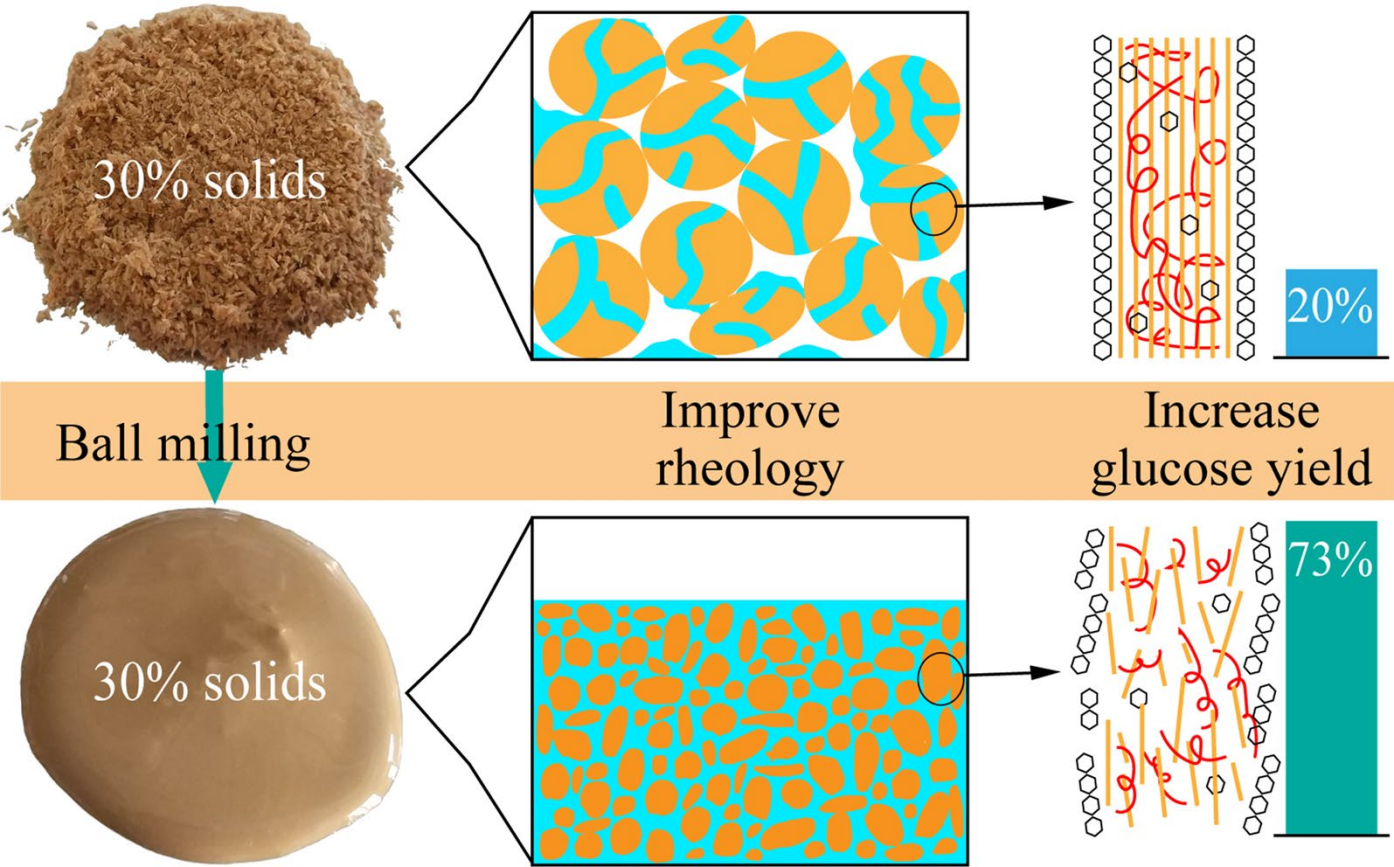
Schematic diagram of the effect of ball milling on the rheological properties and digestibility

The sugar yield of BMCS under different enzyme loading and different solids loading is shown in Fig. 4a. Results show that increasing milling time could increase the sugar yield under different solids loading. In specific, at enzyme loading of 10 FPU g^−1^ solids and solids loading of 10%, the glucose yield increased from 19.9% for BMCS0 to 57.3% for BMCS30, and further increased to 72.6% for BMCS60. Further increasing milling time to 120 min, glucose yield only increased to 77.1%, suggesting that there is little value to mill for more than 60 min, as ball milling is a high energy consumption process. In addition, we found that the enzymatic hydrolysate contained considerable cellobiose, indicating that the fermentable monosaccharides can be further improved by increasing the proportion of related enzymes. The increase of glucose yield may be a combination of the increase of specific surface area (SSA), the decrease in cellulose crystallinity and degree of polymerization (DP), and the dissociation of the cross-linked cellulose–hemicellulose–lignin complex after ball milling [21, 22, 31]. The increase of SSA, the decrease of DP, and the dissociation of the combination between lignin and carbohydrates expose more reactive site for enzyme to hydrolysis, and the loose disordered structure of amorphous cellulose is more active, thus boosts the EH of corn stover. The positive effect of ball milling on the rheological behavior and enzymatic hydrolysis of corn stover slurries could be explained using a schematic diagram shown in Fig. 5.

As mentioned above, cost-effective distillation requires an ethanol concentration above 4% (w/w), i.e., fermentable sugar concentration higher than 8% (w/w) (corresponding to around 87 g L^−1^). It can be seen from Fig. 4b that the total monomeric sugar concentration for BMCS30/BMCS60/BMCS120 at 30% solids loading is still higher than 87 g L^−1^ after reducing enzyme loading from 10 FPU g^−1^ solids to 5 FPU g^−1^ solids, while for 20% solids loading, the results are different. After reducing the enzyme loading, the glucose yield of BMCS30 and BMCS60 at 20% solids loading decreases from 57.3% and 72.6% to 51.4% and 58.3%, respectively, but the decrease of yield is less for 30% solids loading. Future work should be carried out to optimize the enzyme loading and enzyme ratio (e.g., add xylanase) to further increase the sugars’ yield.

**Fig. 6:**
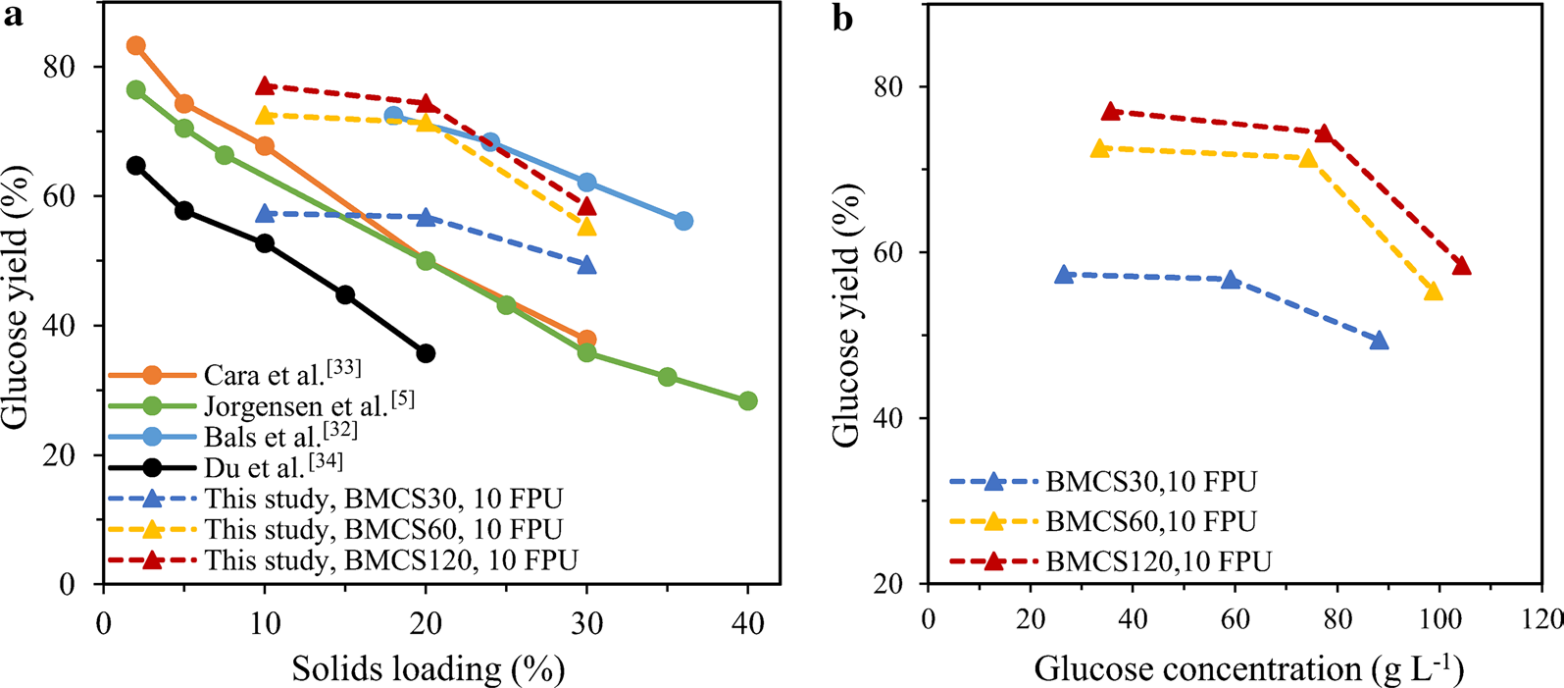
Effect of solids loading and glucose concentration on glucose yield. **a** Solids loading (data collected from several publications) and **b** glucose concentration

The change of glucose yield with solids loading is shown in Fig. 6a. It can be seen from the figure that the so-called ‘solids effect’, that is, the sugar yield decrease at high-solids loading, has been demonstrated in previous reports on high-solids EH of different pretreated lignocellulosic substrates [5, 32–34]. While in this study, the glucose yield of BMCS was basically unchanged when the solids loading increased from 10 to 20%, and further increased the solids loading to 30%, the glucose yield was significantly reduced. These results indicate that ball milling can raise the threshold of solids effect, in other words, alleviate the solids effect to some extent. The glucose yield appears to begin to decrease when the glucose concentration exceeds 60 g L^−1^ (Fig. 6b), so the solids effect seems to be caused by end-product inhibition. Future works will be carried out to apply the simultaneous saccharification and fermentation (SSF) to this high-solid process to alleviate the reduction of sugar yield caused by product inhibition.

The EH kinetic results show that the glucose yield of BMCS reached a maximum after 10 h (Additional file 1: Figure S2), which is meaningful for shortening the production time of cellulosic ethanol. In addition, inhibitors such as furfural, HMF and acetic acid were not detected during the sugar analysis. Conventional thermochemical or physicochemical pretreatment may produce these inhibitors [35]. Running at high-solids potentially implies increased concentration of these inhibitors and thus severely affects the fermentability of the microorganisms. Detoxification by overliming, ion exchange, zeolites or laccase enzyme is usually required before saccharification [36], or a rinsing step followed by dehydration is applied to eliminate the effects of inhibitors, which makes the entire process costly and some soluble sugar might be washed away. The production of a high-concentration sugar syrup free of fermentation inhibitors is very attractive to the downstream fermentation and distillation process.

## Conclusions

Ball milling significantly modified the physicochemical properties of corn stover. The dramatic decrease of particle size leaded to a decrease of entanglement between particles, and the increase of free water enhanced the lubrication; these two factors significantly reduce the viscosity and yield stress of BMCS slurries. As a result, ball milling allowed corn stover to successfully hydrolyze at up to 30% solids loading in a shake flask and maintain good mixing. Ball milling significantly decreased the crystallinity and DP of corn stover, and dissociated the cross-linked cellulose–hemicellulose–lignin complex, thus boosted the glucose yield at high-solids EH, and the resultant hydrolysate was demonstrated to contain fermentable sugar concentration exceeding 87 g L^−1^ (minimum sugars’ concentration for cost-effective ethanol distillation), and no toxic fermentation inhibitors were detected. Furthermore, the energy balance analysis demonstrated that the increased energy used for ball milling could be balanced by the reduced mixing energy.

## Methods

### Corn stover and enzyme preparation

The corn stover used in this study was collected from Shangzhuang Experimental Station of China Agricultural University. The whole crop residues were air-dried and then cut into 2–3 cm length before drying at 40 °C for 48 h, and finally milled to pass through a 1-mm screen using an RT-34 milling machine (Hongquan Pharmaceutical Machinery Ltd., Hong Kong, China). The sample obtained here was denoted as BMCS0.

The enzyme preparation used in this study was Novozymes Cellic CTec2, which was purchased from Sigma-Aldrich (St. Louis, MO, USA). The filter paper activity of CTec2 was determined to be 160.8 FPU mL^−1^. The protein content of CTec2 was measured by the Bradford method using bovine serum albumin as a standard [37], and the protein content was determined to be 106.2 mg mL^−1^.

### Ball milling

The milling was conducted in a vibratory ball mill machine equipped with 2-L ZrO_2_ chamber (CJM-SY-B, Qinhuangdao Taiji Ring Nano Ltd., Hebei, China). The BMCS0 sample was mixed with ZrO_2_ ball (6–10 mm diameter) with a volume ratio of 1:2 and a filling rate of 30%, then milled for 10, 20, 30, 60 and 120 min, the resulting powders were denoted as BMCS10, BMCS20, BMCS30, BMCS60 and BMCS120, respectively. During the ball milling process, the temperature was controlled below 20 °C using a cooling system.

### Characterization of physicochemical properties

#### Composition analysis

The composition of BMCS was measured by a two-step acid hydrolysis method according to National Renewable Energy Laboratory (NREL) standard analysis procedure [38].

#### Distribution of particle size

A laser scattering particle size analyzer, Model Mastersizer 3000 (Malvern Instruments Ltd., United Kingdom), was used to measure the particle size distribution of BMCS by dry mode. The median particle size (*D*_*50*_) was regarded as the average particle size of the sample.

#### Porous structure

The porous structure of the BMCS, such as pore volume (PV) and porosity were measured by an AutoPore-9500 mercury porosimeter (Micromeritics Instrument Ltd., United States) using a powder sample tube with the pressure ranges from 0.52 Pisa to 60,000 Pisa (corresponding to a pore size range of 347,263–3 nm) and an equilibrium time of 10 min.

#### X-ray diffraction

The X-ray diffraction (XRD) pattern was obtained by a Bruker D8 advance X-ray diffractometer (Bruker AXS Inc., WI, Germany) with a Cu Kα radiation source operated at 40 kV and 40 mA. The scanning range of *2θ* was from 5° to 40°, with a scanning speed of 2° min^−1^ and a step size of 0.02°. The crystallinity index (CrI) of BMCS was determined by the peak height method and the formula was as follows [39]:

$$ {\text{CrI}} = \left( {I_{002} - I_{\text{am}} } \right)/I_{002} \times 100, $$

where *I*_002_ is the intensity of [002] peak at approximately *2θ* = 22.5°, and *I*_am_ the intensity of the minimum between the [002] and the [101] peaks at around *2θ* = 18°.

### High-solids enzymatic hydrolysis

Commercial enzyme preparation Novozymes Cellic CTec2 was used for high-solids EH of BMCS. The digestion was carried out in a 250-mL shake flask loaded with 60 g BMCS slurry with 10%, 20% and 30% solids content prepared by citrate buffer (pH 4.8) using an enzyme loading of 5 and 10 FPU g^−1^ solids, and tetracycline hydrochloride (0.08 g L^−1^) was added to avoid microbial interference. EH was performed in a shaking water bath operating at 150 rpm and 50 °C.

After digestion for 2 h, 5 h, 10 h, 24 h, 48 h and 72 h, the well-mixed solid–liquid mixture was taken for rheological measurement and products’ analysis. Part of the samples was subjected to apparent viscosity measurement to estimate the energy consumption of stirring during hydrolysis process. The sugars and byproducts analysis was based on NREL standard analysis procedure [40]; deducting the sugar concentration in the enzyme blank to obtain the final result. Sugar yield was defined as the percentage of monosaccharide released during digestion process based on the theoretical maximum.

### Rheological measurement

The rheological measurement of corn stover slurries before and after enzymatic hydrolysis was carried out with an AR-G2 rheometer (TA Instruments) using a 25-mm serrated parallel plate geometry with a 1.5 mm gap. Plate temperature was set to 50 °C in all cases.

The apparent viscosity of slurries was measured using flow sweep mode. The transducers were initialized (Conditioning Transducer) before data acquisition, ensuring that the normal force and torque transducer were in Force Rebalance Transducer (FRT) mode, the appropriate torque range was selected, and the normal force and torque were zeroed. The shear rate was logarithmically increased from 0.01 to 100 s^−1^. All measurements were carried out in, at least, triplicates with fresh samples.

Unlike model extrapolation method [14], the yield stress reported here was measured using an AR-G2 rheometer in oscillatory amplitude mode. Similarly, the transducers were initialized (Conditioning Transducer) before data acquisition. The strain amplitude was logarithmically increased from 0.01 to 100% at a frequency of 1 Hz. The yield stress (*τ*_*y*_) was calculated as the maximum value of *τ* = *G*^’^*γ, where *G*^*’*^ is the elastic modulus of the slurry, and *γ* the strain amplitude [18]. All measurements were carried out in, at least, triplicates with fresh samples.

### Calculation of energy consumption for ball milling and stirring

The energy consumption of ball milling was measured by a wattmeter (Yadu, Ltd., Shanghai, China). The wattmeter recorded the real-time power of the ball mill machine every second, and the energy consumption was calculated by integrating the recorded power over milling time:

$$ {\text{EM}} = \frac{{\mathop \smallint \nolimits_{0}^{t} P_{t} {\text{d}}t}}{m}, $$

where EM (kW h kg^−1^) is the energy consumption of ball milling, and converted to MJ kg^−1^ with a coefficient of 3.6; *P*_*t*_ (kW) is the power of milling machine at time t; *m* (kg) is the mass of corn stover.

In a specific stirring tank, the energy dissipated by the impeller depends on the viscosity of the slurry when operating in the laminar and transition region (Reynolds number Re < 10^4^) [26]. More specifically, the power consumption *P* = *N*_p_*ρ*N^3^*D^*5*^, where *N*_*p*_ is the impeller power number, *ρ* the fluid density, *N* the impeller speed, and *D* the impeller diameter. And *N*_*p*_ is a function of Reynolds number (*N*_*p*_ = K*Re^−*1*^ = K*η_*a*_/(ρ*N*D^*2*^*)*) [41], thus *P* = K*η_a_*N^2^*D^*3*^, where *K* is power constant; therefore, the power consumption for mixing is proportional to the apparent viscosity of the slurry at a specific stirred tank and stirring speed. Assumed that the EH was carried out in a 7-L conventional stirred tank equipped with a helical ribbon impeller (*D* = 0.185 m) [42], then the energy consumption for mixing during EH could be estimated as described below. To save energy, the stirring speed is relatively low at high-solids EH, for example, the stirring speed in the pilot scale reactors of the NREL is 55 rpm [14]. The corresponding effective shear rate is calculated by *γ*_eff_ = *K*_s_* N [43], where *K*_*s*_ is the Metzner constant and *K*_*s*_ equal to 32.9 for the selected helical ribbon impeller [42]. Therefore, selected the apparent viscosity at *γ*_eff_ = 32.9*55/60 = 30.16 s^−1^ (according to the data exported from the instrument, data at 25.12 s^−1^ were chosen). According to the power consumption curve of helical ribbon impeller, the power constant (*K*) was taken as 173.1 [42]. So far, the power consumption for stirring at different hydrolysis time could be estimated by *P* = K*η_a_*N^2^*D^*3*^, and the energy consumption during the whole EH period (48 h) was calculated by integrating over time.

## Supplementary information

**Additional file 1: Figure S1.** Apparent viscosity as a function of shear rate and shear stress for BMCS slurry at different solids loading. **Figure S2.** Enzymatic hydrolysis kinetic data of ball-milled corn stover. **Table S1.** Free water amount for BMCS slurry at 30% solids loading.

## Data Availability

All data generated or analyzed during this study are included in this published article.

## Abbreviations

EH: Enzymatic hydrolysis
BMCS: Ball-milled corn stover
SEM: Scanning electron microscope
XRD: X-ray diffraction
CrI: Crystallinity index
PV: Pore volume
SSA: Specific surface area
DP: Degree of polymerization
