# Supplementary material for: High-solids enzymatic hydrolysis of ball-milled corn stover with reduced slurry viscosity and improved sugar yields
Source: Biotechnol Biofuels. 2020 Apr 20;13:77. doi: 10.1186/s13068-020-01717-9 (PMC7171840; doi:10.1186/s13068-020-01717-9)
Supplement: Supplementary file 1 — Additional file 1: Figure S1. Apparent viscosity as a function of shear rate and shear stress for BMCS slurry at different solids loading. Figure S2. Enzymatic hydrolysis kinetic data of ball-milled corn stover. Table S1. Free water amount for BMCS slurry at 30% solids loading. [file 13068_2020_1717_MOESM1_ESM.docx]

**High-solids enzymatic hydrolysis of ball-milled corn stover with reduced slurry viscosity and improved sugar yields**

Minsheng Lu^†^, Junbao Li^†^, Lujia Han^†^, Weihua Xiao^†, *^

^†^ College of Engineering, China Agricultural University (East campus), 17 Qing-Hua-Dong-Lu, Hai-Dian District, Beijing, 100083, P.R. China

**^*^** Corresponding author: Dr. Weihua Xiao,

P.O. Box 191, College of Engineering,

China Agricultural University (East campus),

17 Qing-Hua-Dong-Lu, Haidian district,

Beijing 100083, P. R. China.

Telephone: 86-10-62736778, Fax: 86-10-62736778

Email: xwhddd@163.com

**Figure S1.** **Apparent viscosity as a function of shear rate and shear stress at different solids loading.** (a) BMCS0, (b) BMCS10, (c) BMCS20, (d) BMCS30, (e) BMCS60 and (f) BMCS120 at different solids loading.

**
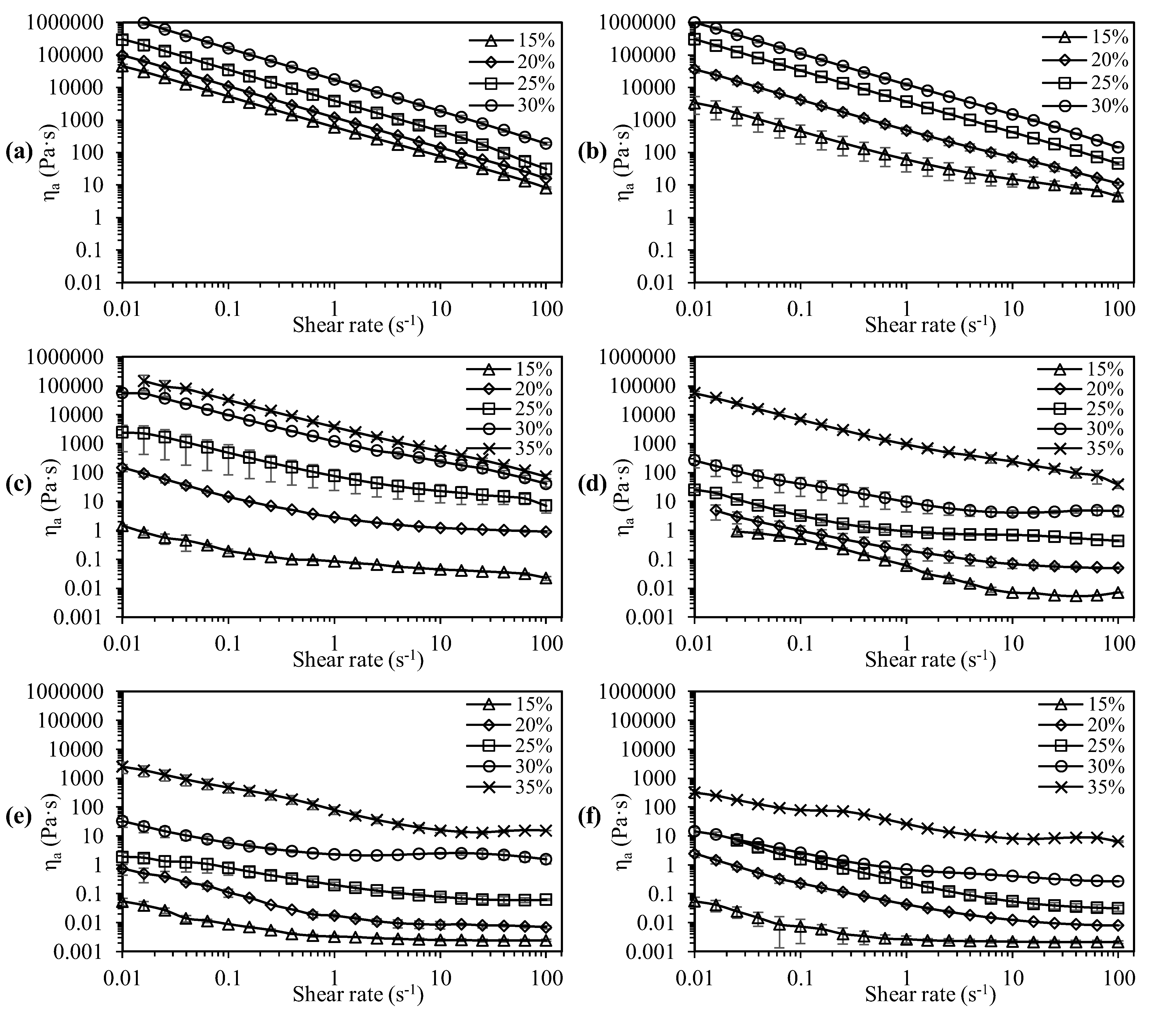
**

**
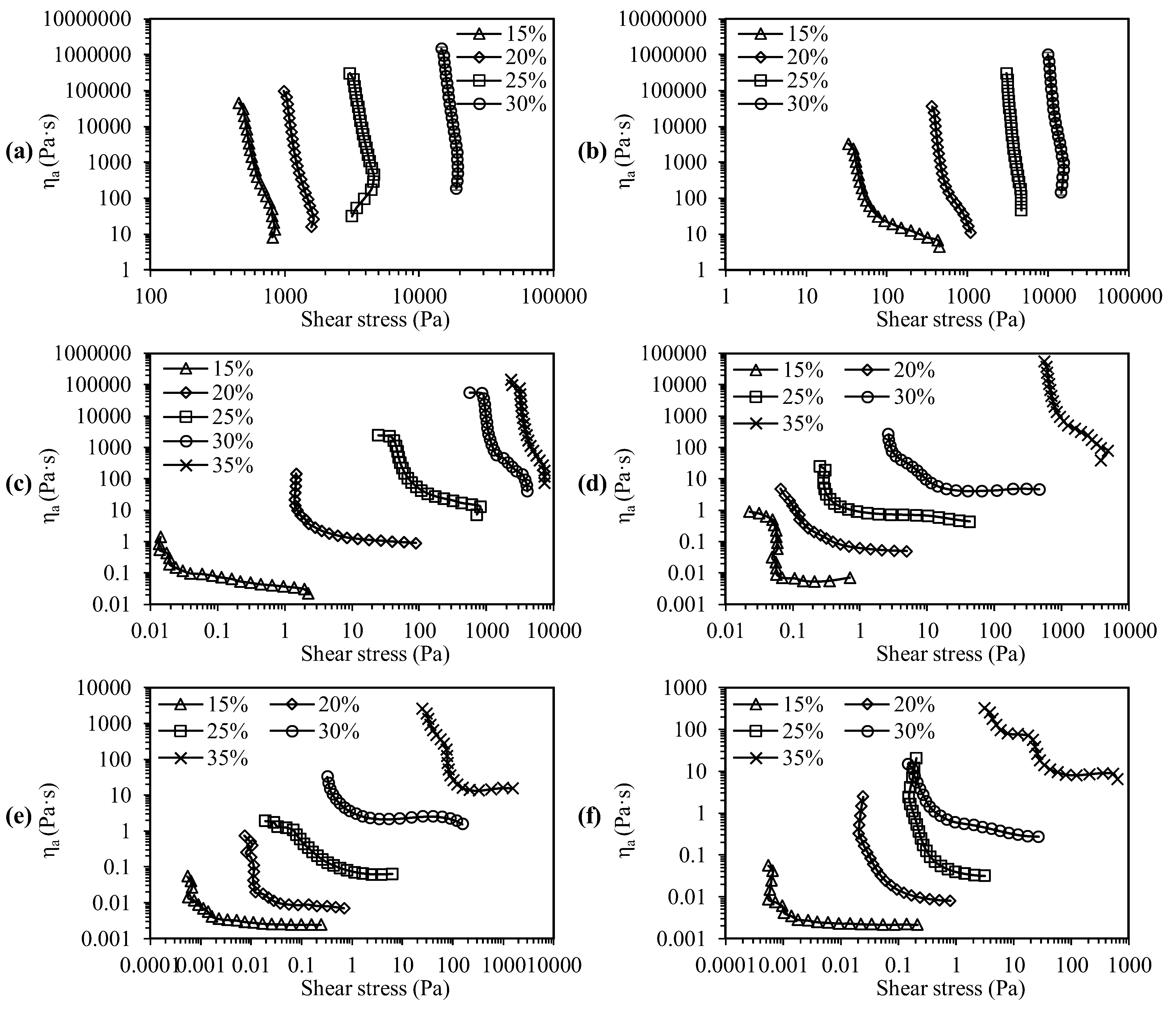
**

**Figure S2. The enzymatic hydrolysis kinetic data of ball-milled corn stover using 10 FPU/g solids at different solids loading**. (a) glucose yield and (b) xylose yield.

**Table S1 Free water amount for BMCS slurry at 30% solids loading.**

| Sample | Porosity (%) | Bulk density (g cm^-3^) | Free water (g g^-1^ slurry) |
| --- | --- | --- | --- |
| BMCS0 | 78.88 | 0.295 | -0.102 |
| BMCS10 | 76.96 | 0.318 | -0.026 |
| BMCS20 | 71.62 | 0.393 | 0.153 |
| BMCS30 | 62.05 | 0.523 | 0.344 |
| BMCS60 | 56.55 | 0.597 | 0.416 |
| BMCS120 | 56.22 | 0.602 | 0.420 |

The free water amount (*m_fw_*) is calculated based on porosity measurement: $\text{m}_{\text{fw}}\text{= }\text{m}_{\text{w}}\text{ – }\frac{\text{m}_{\text{s}}}{\text{ρ}_{\text{b}}}\text{ × porosity × }\text{ρ}_{\text{w}}$, where *m_fw_* is the free water amount (g g^-1^ slurry), *m_w_* and *m_s_* is the mass of total water and solids (g g^-1^ slurry), respectively, *ρ_b_* is the bulk density of corn stover (g cm^-3^), *ρ_w_* is the density of water (g cm^-3^). The negative value of free water amount might be caused by the fact that these is more pore volume than liquid at high solids loading, or due to the overestimated porosity by the mercury intrusion porosimetry.
